# Proteomic profile of human spermatozoa in healthy and asthenozoospermic individuals

**DOI:** 10.1186/s12958-018-0334-1

**Published:** 2018-02-27

**Authors:** Xiaodan Cao, Yun Cui, Xiaoxia Zhang, Jiangtao Lou, Jun Zhou, Huafeng Bei, Renxiong Wei

**Affiliations:** Department of Clinical Laboratory, Ningbo Municipal Hospital of Traditional Chinese Medicine, Ningbo, 315000 China

**Keywords:** Proteome, Spermatozoa, Sperm motility, Asthenozoospermia, Infertility

## Abstract

Asthenozoospermia is considered as a common cause of male infertility and characterized by reduced sperm motility. However, the molecular mechanism that impairs sperm motility remains unknown in most cases. In the present review, we briefly reviewed the proteome of spermatozoa and seminal plasma in asthenozoospermia and considered post-translational modifications in spermatozoa of asthenozoospermia. The reduction of sperm motility in asthenozoospermic patients had been attributed to factors, for instance, energy metabolism dysfunction or structural defects in the sperm-tail protein components and the differential proteins potentially involved in sperm motility such as COX6B, ODF, TUBB2B were described. Comparative proteomic analysis open a window to discover the potential pathogenic mechanisms of asthenozoospermia and the biomarkers with clinical significance.

## Background

Infertility is defined as the lack of ability to achieve a clinical pregnancy after one year or more of unprotected and well-timed intercourse with the same partner [1]. It is estimated that around 15% of couples of reproductive age present with infertility, and about half of the infertility is associated with male partner [2, 3]. With the continuing world-wide increase in male infertility, it has become a major health problem attracting more clinical attention. Asthenozoospermia(AS) is a common cause of human male infertility characterized by reduced sperm motility with sperm motility< 50% or progressive motility< 25% [4]. The proteins involved in the normal physiology of the sperm motility is rather scarce and the molecular basis of asthenozoospermia is not yet fully understood [5–7]. The causes of poor sperm motility include abnormal metabolism in the testicular tissue or epididymis, structural deficiency in the sperm tail, and functional deficiency of the epididymis or other accessory sex glands [8–10]. Routine semen analysis on sperm motility is only a clinical indicator of male fertility and does not account for the underlying cause of defects associated with sperm movement. It is possible that individual protein defects in spermatozoa might cause fertilization failure [4] and it has become clear that identifying the precise proteins and the pathways involved in sperm motility is needed [5].

## Application of proteomic techniques in male infertility

**Fig. 1:**
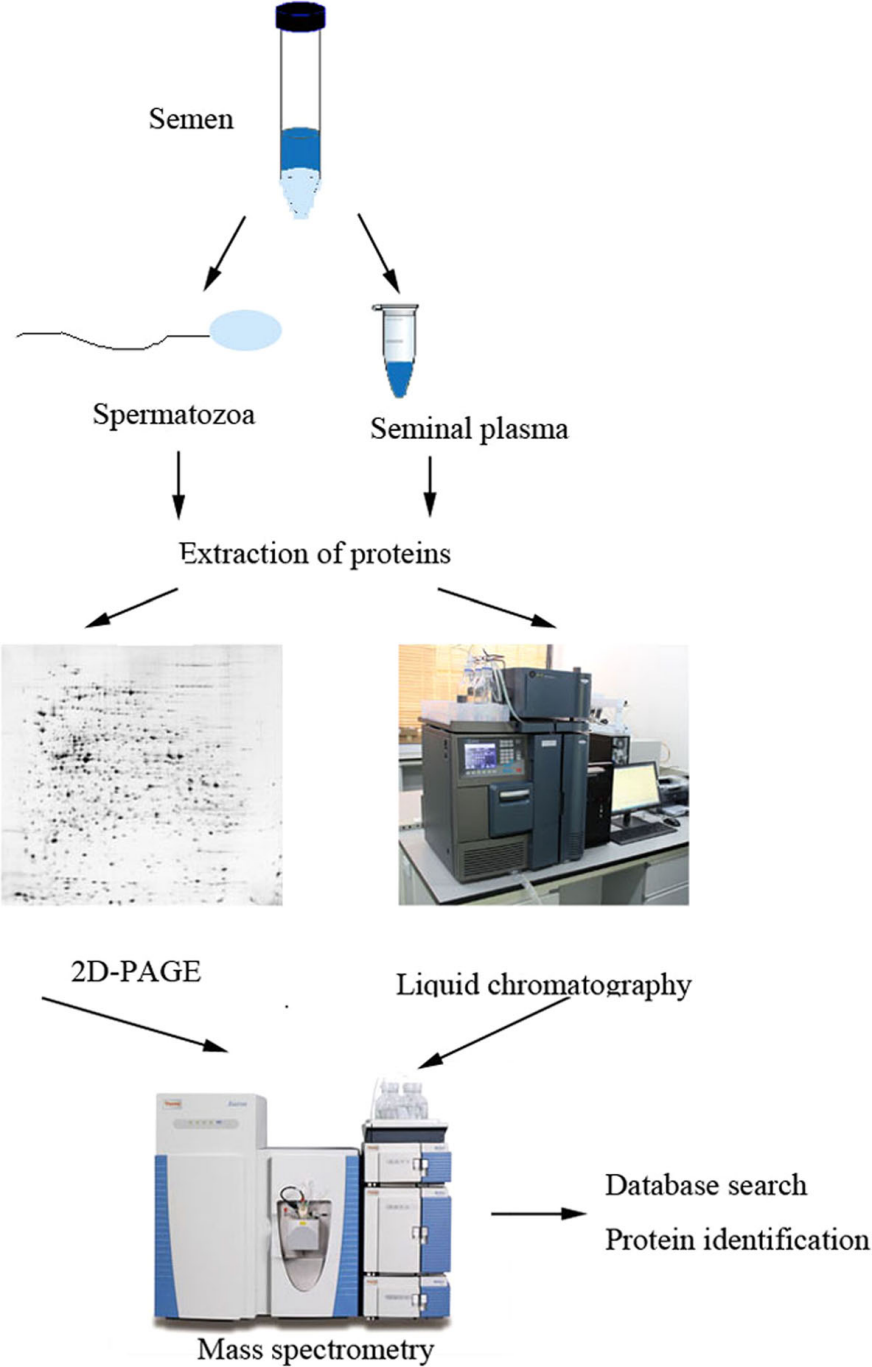
Schematic diagram showing the proteomic analysis of the sperm cells and seminal plasma. After collection and liquefaction of ejaculated semen, the desired target components are purified through density gradients centrifugation. Proteins are extracted and separated by two-dimensional polyacrylamide gel electrophoresis (2D–PAGE) or liquid chromatography(LC), and then followed by identification using mass spectrometry and database search

Proteomic approaches, such as two-dimensional (2D) polyacrylamide gel electrophoresis, mass spectrometry (MS), matrix-assisted laser desorption ionization time of flight (MALDI-TOF) and isobaric tags for relative and absolute quantitation (iTRAQ) could be useful in identifying a wide range of the proteins responsible for diagnosis of sperm dysfunctions and the regulatory mechanism of male fertility [11–17] (Fig. 1). A number of studies have utilized high-throughput techniques to study protein alterations in fertile versus infertile groups, for example, normal versus malformed, capacitated versus incapacitated and low versus high sperm motility [11, 18–20]. These techniques offers the comprehensive understanding of sperm proteins with their particular structure and also provides a new view to study different functional states of sperm [21, 22]. For example, Chan et al. performed MADLI-TOF to address altered protein phosphorylation and aberrant sperm motility and 12 proteins were identified [18]. Secciani et al. employed MS/MS approach to analyze the protein profiles of capacitated versus ejaculated spermatozoa [23]. Sperm protein expression in asthenozoospermic patients and normozoospermic controls were compared, and several quantitative and qualitative significant variations were found [15]. Some of these differential proteins may be potential target proteins that could benefit further research on sperm function-related infertility and also shedding light on the key proteins involved in fertilization.

## Spermatozoal proteome in asthenozoospermia

Sperm motility is an important prerequisite for successful fertilization. The proteomic studies on asthenozoospermic individuals are increasing and more and more proteins and pathways involved in sperm motility are identified (Table 1, Table 2). Hashemitabar et al. [24] investigated the protein expression profiles of human sperm tail from asthenozoospermic and normozoospermic individuals using the MALDI-TOF-TOF approach. Among the fourteen differentially expressed proteins, eleven proteins [(A-kinase anchor protein 4 (AKAP4), outer dense fiber 2 (ODF2), tubulin beta 2B (TUBB2B), cytochrome c oxidase subunit 6B (COX6B), glutathione S-transferase Mu 3 (GSTMu3), phospholipid hydroperoxide glutathione peroxidase (PHGPx), glyceraldehyde-3-phosphate dehydrogenase, testis-specific (GAPD-S), voltage-dependent anion-selective channel protein 2 (VDAC2), heat shock-related 70 kDa protein 2 (HSPA2), stress-70 protein, mitochondrial (HSPA9), Sperm protein associated with the nucleus on the X chromosome B (SPANXB)] had increased amounts in normal controls, and three proteins [clusterin (CLU), keratin, type II cytoskeletal 1 (KRT1), isoaspartyl peptidase/L-asparaginase (ASRGL1)] had higher expression levels in asthenozoospermic samples. In the functional categorization, the majority fall into five groups: energy and metabolism (COX6B, GAPDS, PHGPx), movement and structural organization (TUBB2B, ODF2, AKAP4, KRT1, CLU), stress response and turn over (HSPA2, HSPA9), signaling and transport (VDAC2), and antioxidant activity (GSTMu3).

**Table 1:** Sperm proteins with a significantly higher or lower expression in asthenozoospermia in different proteomic studies

Study	Method	Identified proteins	
		up-regulated in astheno	down-regulated in astheno
Hashemitabar et al.	2D electrophoresis MALDI-TOF-TOF analysis	CLU, KRT1, ASRGL1	AKAP4, ODF2, TUBB2B, COX6B, GSTMu3, PHGPx, GAPD-S, VDAC2, HSPA2, HSPA9 and SPANXB
Siva et al.	2D PAGE MALDI MS/MS analysis	TRIS, GPK2, SCOT1, unidentified	TUBB2C, tektin 1, PSMA3, HSPA2
Martı’nez-Heredia et al.	2D electrophoresis MS analysis	CLUpre, DLDpre, FHpre, HSPA2, IMPA1, PSMB3, SEMG1pre, TEX12, MPST/ECH1pre	ACTB, ANXA5, COX6B, histone H2A, PIP, PIPpre, calcium binding protein-S100A9
Shen et al.	2D electrophoresis MALDI-TOF analysis	PIPpre, flagellin, GPX4, GAPDH	TEKT4, Lacto, ODF2, SPANXB, PGK2, GRP78, Hsp70, DJ-1, CAB2, Actin, Heat shock protein 70 testis variant
Zhao et al.	2D electrophoresis MALDI-TOF analysis	PGM2, TPI, GOT-1, ODF, GS, semenogelin I precursor	IDH-α, CA-II, GDI-1, MSS1

**Table 2:** Summary of Gene Ontology(GO) analysis of differential sperm proteins in asthenozoospermia in different proteomic studies

Study	GO term	Protein names
Hashemitabar et al.	energy and metabolism	COX6B, GAPDS, PHGPx
	movement and structural organization	TUBB2B, ODF2, AKAP4, KRT1, CLU
	stress response and turn over	HSPA2, HSPA9
	signaling and transport	VDAC2
	antioxidant activity	GSTMu3
Siva et al.	energy and metabolism	TRIS, GPK2, SCOT1, unidentified
	movement and organization	TUBB2C, tektin 1
	protein turnover, folding and stress response	PSMA3, HSPA2
Martı’nez-Heredia et al.	energy production	COX6B, DLDpre, Fhpre, ECH1pre
	structure and movement	ACTB, H2A, PIP, PIPpre, SEMG1
	cell signalling and regulation	ANXA5, S100A9, IMPA1
Zhao et al.	metabolic enzymes	IDH-α, PGM2, TPI, GOT-1, CA-II
	structure	GDI-1,ODF
	other	14-kDa fragment and 17-kDa fragments of semenogelin I

Proteins responsible for energy and metabolism (COX6B, GAPDS and PHGPx), associated with the production of ATP in distal regions of the flagellum [25, 26], had a decreased expression in asthenozoospermic sperm samples. The decreased level might affect the structure of fibrous sheath and ATP production, which might impair sperm motility. VDAC family proteins with an ATP binding site regulates mitochondrial outer membrane permeabilization to ions and ATP molecules [27, 28] and knockout of the VDAC2 and VDAC3 gene resulted in significantly reduced sperm motility and embryo development[29], suggesting that VDACs are indispensable for sperm energy metabolism and successful fertilization.

Sperm is a highly polarized cell and its motility is fully dependent on flagellum. The ability of sperm to move forward is crucial for the successful fertilization of an egg [30]. The reduction of sperm motility in asthenozoospermic men has been attributed to factors such as structural defects in the sperm-tail protein components [8]. Sperm flagellum consists of an axoneme surrounded in distinct regions by accessory structures such as the fibrous sheath and ODF. AKAP3 and AKAP4 are the most abundant structural proteins in the fibrous sheath and have anchoring sites for cyclic-AMP-(cAMP)-dependent protein kinase that increases tyrosine phosphorylation of sperm proteins and regulating flagellar function [31]. Deleting AKAP4 gene in male mice caused dysplasia of the fibrous sheath (DFS) which lead to defective sperm motility [32]. Absence of AKAP4 resulted in loss of effective sperm motility because cAMP-dependent signaling processes fail to be associated with the fibrous sheath [33]. The fibrous sheath is involved in regulating sperm maturation, motility and capacitation and AKAP4 could influence the sperm locomotion and might be a biomarker of understanding prefertilization events. Numerous polypeptides such as ODF1 and ODF2 constitute the ODF sheath which help to preserve the elastic rigidity of sperm flagellum [34, 35]. An ultra structural study of asthenozoospermia individuals with DFS showed the abnormal extension of ODF to the principal piece[36]. Reduction of one or more ODF proteins might result in decreased elasticity of sperm flagellum and cause nonfunctional tails [37]. There is a possibility for a screening regarding the distribution of sperm tail proteins related to motility disorder.

Tubulin is known to play a key role in the formation of microtubules of flagellum. TUBB2C is a major cytoskeletal protein present in the head and flagellum of spermatozoa and existed actual influences on sperm motility [38, 39]. TUBB2B, expressed in post-mitotic cells in male germ cells, are essential for the the formation of meiotic spindles and cytoplasmic microtubules and also share in the organization of 9 + 2 of the axonemal microtubule in sperm tail [40]. Sperm in asthenozoospermic patients were defective in motility might due to the lower expression of tubulin with structural defects in sperm flagellum like dysplasia of the fibrous sheath (DFS) with missing axonemal central pairs and complete distortion of 9 + 2 axonemal structure [36].

HSP family proteins are stress response proteins which protect the cell against oxidative stress and pathological changes [41]. HSPA9 is predominant in the mitochondrial matrix and required during protein translocation into mitochondria and the refolding of mitochondria proteins [42]. GSTMu3 is a kind of antioxidant proteins and participates in the detoxification of toxins, drugs and oxidative stress products [43]. The down-regulated of HSPA9 and GSTMu3 in asthenozoospermia could lead to the accumulation of oxidative stress products and impair sperm motility.

Siva et al. [44] employed 2D PAGE MALDI MS/MS analysis to compare the sperm proteome of asthenozoospermic and normozoospermic patients. Eight proteins showing changes in levels between two groups were revealed and the differential proteins belonged to three functional groups: energy and metabolism [triose-phosphate isomerase(TRIS), glycerol kinase, testis specific 2 (GPK2) and succinyl-CoA:3-ketoacid CoA transferase 1 (SCOT1), unidentified], movement and organization [tubulin beta-2C chain(TUBB2C) and tektin 1] and protein turnover, folding and stress response [proteasome subunit alpha type 3 (PSMA3) and HSPA2]. It was interesting to note that proteins falling in the ‘energy and metabolism’ group are higher in asthenozoospermic individuals and the high expression may be because of the redundant cytoplasm (droplets) contained in immaturation spermatozoa, while proteins with altered intensity in other two functional groups are higher in normozoospermic donors. TUBB2C, TPIS, HSPA2 and PSMA3 have been reported to get S-nitrosylated in human sperm during capacitation [17], indicating an involvement of these proteins in various sperm functions associated with capacitation, including sperm motility. HSPA2 has been shown to be a marker for sperm maturity [45] with lower amounts in oligoteratozoospermic men [46].

Proteasomes have been detected in sperm from various species and the major function of proteasome is to degrade nuclear and cytosolic ubiquitinated proteins [47–49]. Its low expression in asthenozoospermia might lead to the accumulation of higher ubiquitinated molecules and was related to sperm motility [48]. Studies on the sperm proteome from asthenozoospermic samples have revealed that the relative expression of some component of the proteasome complex is different. Zhao et al. [15] reported that the 26S protease regulatory subunit 7 (MSS1) is decreased in asthenozoospermic patient, in contrast to the study by Martı’nez-Heredia et al. [50] which showed that PSMB3 is increased in asthenozoospermia. The different trend for proteasome subunit is not clear and may be attributed to ethnic differences (studies by Siva and Zhao group worked on Asian population and Martı’nez-Heredia et al., worked on European population). Further study on a larger sample size would be required to confirm these findings.

Martı’nez-Heredia et al. [50] compared the expression of asthenozoospermic samples to that of semen donor controls using two-dimensional proteomic analysis and seventeen protein spots have been identified at different amounts. Of these proteins identified, COX6B, dihydrolipoamide dehydrogenase precursor (DLDpre), fumarate hydratase precursor (FHpre) and 3-mercapto-pyruvate sulfurtransferase/dienoyl-CoA isomerase precursor (MPST/ECH1pre) constitute the ‘energy production’ group; cytoskeletal actin-B (ACTB), histone H2A, prolactin-inducible protein (PIP), prolactin-inducible protein precursor (PIPpre), and semenogelin 1 precursor (SEMG1pre) constitute ‘structure and movement’ group; and the ‘cell signalling and regulation’ group is constituted by annexin-A5 (ANXA5), calcium binding protein-S100A9, and inositol-1 monophosphatase (IMPA1). It is interesting to note that six of these identified proteins are precursor forms [(DLDpre, FHpre, ECH1pre, PIPpre, SEMG1pre and clusterin precursor (CLUpre)] of mature proteins, which could suggest the presence of a generalized post-translation processing problem of these proteins. The accumulation of precursors in asthenozoospermic patients also might lead to the deregulation of some downstream proteins. DLDpre and Fhpre, associated with the major sources of ATP production in sperm, were upregulated in asthenozoospermic patients, which could be involved with a potential decrease in the mature protein and the failure of tail movement of sperm in asthenozoospermia [51, 52]. The results together with those of Zhao et al. [15] demonstrate the existence of a general deregulation in the pathways involved in energy and metabolism in asthenozoospermic patients.

PIP is a 17-kDa glycoprotein present in human seminal plasma. Its aspartyl-proteinase nature suggests its specificity to fibronectin that is one of the major protein constituents of the seminal coagulum [53–55], which indicates that PIP could contribute to the specifical degradation of fibronectin during liquefaction. Lower expression of PIP in asthenozoospermia might result in an incomplete liquefaction of the ejaculate and make a restriction for the movement of spermatozoa.

2D electrophoresis MALDI-TOF analysis was performed to compare sperm protein composition in asthenozoospermic patients with that of normozoospermic samples [15, 56]. Sixteen differentially expressed protein were identified by Shen et al. and tektin 4 (TEKT4) and ODF2 appeared only in normozoospermic samples [56]. Tektins and ODFs form the main components of the cytoskeleton in sperm flagella, and the reduction of sperm motility in asthenozoospermic men has been attributed to factors such as abnormal expression of the structural constituents of cytoskeleton [8]. 78 kDa glucose-regulated protein (GRP78), belonging to HSP protein located on endoplasmic reticulum(ER), is down-regulated in asthenozoospermic samples. GRP78 was able to bind to the acrosomal region of capacitated human spermatozoa and to modulate the binding interaction between sperm and the zona pellucida, which might affect intracellular signaling transduction and the capacitation of sperm [57, 58].

Ten differentially expressed proteins involved in the regulation of sperm motility were identified by Zhao et al. and these proteins were distributed into three categories: metabolic enzymes, tructure-associated proteins and other functional proteins [15]. Contrary to the previous studies, ODF protein showed higher expression in asthenozoospermia in this study and the disturbance of ODF protein may be associated with sperm motility defect. Half of the identified proteins are related with ATP metabolism to support sperm movement. The overexpression of phosphoglycerate mutase 2 (PGM2), triosephosphate isomerase(TPI) and glutamate oxaloacetate transaminase-1(GOT-1) in asthenozoospermic patients may be a compensatory reaction to the reduced motility sperm and weakened TCA cycle associated with low isocitrate dehydrogenase subunit α (IDH-α) expression [15].

## Post-translational modifications in spermatozoa of asthenozoospermia

Post-translational modifications especially phosphorylation play an important role in sperm motility and protein phosphorylation is reported to be a prerequisite for spermatozoa to fertilize an egg. Chan et al. [23] performed 2D electrophoresis MALDI-TOF MS approach to analyze motility and protein phosphorylation in healthy and low-motility-sperm and identified twelve spots as having differential phosphorylation. Among these 12 proteins, 10 proteins were hypophosphorylated in asthenozoospermic sperm, while 2 proteins exhibited a relatively lower phosphorylation level in normal sperm. Parte et al. [59] investigated the expression of phosphoproteins in normal- and asthenozoo-sperm by Nano UPLC–MSE and Ingenuity Pathways Analysis to identify the differential phosphoproteins and the key pathways regulating sperm motility. 66 phosphoproteins were significantly different of which 27 proteins were decreased and 39 proteins were increased in asthenozoosperm compared with those in normal samples. The differentially regulated proteins contained the family of HSPs, cytoskeletal proteins and proteins involved in the fibrous sheath and energy metabolism. Pathway analysis indicated that carbohydrate and energy metabolism, cyclic AMP signaling, PI3K/AKT signaling and pathway regulating actin based motility by Rho were significantly altered. The data identified demonstrated that the signature molecules of metabolic enzymes, fibrous sheath associated proteins and cytoskeletal proteins have the potential as biomarkers for diagnosing axonemal defects, a defective fibrous sheath, and abnormal mitochondria in asthenozoospermia.

## Proteome in the seminal plasma of asthenozoospermia

Seminal plasma is a mixture of secretions from the testis, epididymis and several male accessory glands and a growing number of seminal plasma proteins have been shown to be related with sperm motility. To further explore the impact of post-testicular processes on sperm motility, seminal plasma proteins from asthenozoospermia individuals and healthy men were collected and analyzed by LC-MS/MS. 101 differentially expressed proteins were identifed and Chain A, Human Dj-1 with sulfinic acid (DJ-1) was the most downregulated protein in the seminal plasma of asthenozoospermic patients. Most of these proteins enriched in the hydrolase activity, catalytic activity and enzyme regulator activity. In addition, the levels of reactive oxygen species (ROS) were higher in asthenozoospermia men and these data suggest that downregulation of the DJ-1 protein could lead to increased oxidative stress for spermatozoa which would influence the quality of the semen and sperm motility [60]. Saraswat et al. [61] have performed a differential proteomic analysis of the sperm and seminal plasma in normozoospermia and asthenozoospermia samples and identified 667 proteins for label-free analysis in sperm and 429 proteins in seminal plasma. Statistical and mathematical analysis including principal component analysis, OPLS-DA (S-Plot), ROC Curve analysis and self-organizing maps analysis were performed on the dataset. Various pathways were enriched in the proteomic datasets and some of these pathways were gluconeogenesis, glycolysis, stress response, nucleosome assembly, axoneme activation and focal adhesion assembly. The studies elucidated the pathways underlying the sperm motility which can lead to identification of novel treatment avenues.

## Conclusion

In this review, we have compared data of proteome studies from asthenozoospermic men and normal individuals that evaluated the protein profile that controls infertility. Studies compiled in this work would contribute appreciably to the presently limited information available about molecular mechanism underlying sperm motility. There is a need to extend these studies to get a consistent view on the specific proteins involved in asthenozoospermia and their characterization could merit further investigation on sperm motility-related infertility. Ultimately, these findings will contribute towards the development of novel diagnostic markers of male infertility.

## Abbreviations

AKAP4: A-kinase anchor protein 4
ANXA5: annexin-A5
ASRGL1: Isoaspartyl peptidase/L-asparaginase
CAB2: XPA binding protein 2, isoform CRA_b
CA-II: Carbonic anhydrase II
CLU: Clusterin
CLUpre: Clusterin precursor
COX6B: Cytochrome c oxidase subunit 6B
DFS: Dysplasia of the fibrous sheath
DJ-1: Chain A, Human Dj-1 with sulfinic acid
DLDpre: Dihydrolipoamide dehydrogenase precursor
Fhpre: Fumarate hydratase precursor
GAPDH: Glyceraldehyde-3-phosphate dehydrogenase
GAPD-S: Glyceraldehyde-3-phosphate dehydrogenase, testis-specific
GDI-1: Rho GDP-dissociation inhibitor 1
GKP2: Glycerol kinase, testis specific 2
GOT-1: Glutamate oxaloacetate transaminase-1
GPX4: Phospholipid hydroperoxide glutathione peroxidase
GRP78: 78 kDa glucose-regulated protein
GS: Glutamine synthetase
GSTMu3: Glutathione S-transferase Mu 3
HSP: Heat shock protein
HSPA2: Heat shock-related 70 kDa protein 2
HSPA9: Stress-70 protein, mitochondrial
IDH-α: Isocitrate dehydrogenase subunitα
IMPA1: Inositol-1 monophosphatase
iTRAQ: Isobaric tags for relative and absolute quantitation
KRT1: Keratin, type II cytoskeletal 1
Lacto: Lactoferrin
MALDI-TOF: Matrix-assisted laser desorption ionization time of flight
MPST/ECH1pre: 3-mercapto-pyruvate sulfurtransferase/dienoyl-CoA isomerase precursor
MS: Mass spectrometry
MSS1: 26S protease regulatory subunit 7
ODF: Outer dense fiber
PGK2: Phosphoglycerate kinase 2
PGM2: Phosphoglycerate mutase 2
PIP: Prolactin-inducible protein
PIPpre: Prolactin-inducible protein precursor
PSMA: Proteasome subunit alpha
PSMB3: Proteosome subunit beta 3
ROS: Reactive oxygen species
SCOT: Succinyl-CoA:3-ketoacid CoA transferase
SEMG1pre: Semenogelin 1 precursor
SPANXB: Sperm protein associated with the nucleus on the X chromosome B
TEKT4: Tektin 4
TEX12: Testis expressed sequence 12
TPI: Triosephosphate isomerase
TUBB2B: Tubulin beta 2B
TUBB2C: Tubulin beta 2C
VDAC2: Voltage-dependent anion-selective channel protein 2
